# Exploring the use of WeChat for qualitative social research: The case of Italian digital diaspora in Shanghai

**DOI:** 10.3389/fsoc.2023.1144507

**Published:** 2023-02-24

**Authors:** Grazia Moffa, Marco Di Gregorio

**Affiliations:** ^1^Department of Political and Social Studies, University of Salerno, Salerno, Italy; ^2^Department of Culture, Politics and Society, University of Turin, Turin, Italy

**Keywords:** digital migratory space, Italian digital diaspora, digital methods, netnography, qualitative online interviews, WeChat, methodological innovations, social research

## Abstract

The widespread use of digital communication technologies has created new opportunities for social research. In this paper, we explore the limits and potentials of using messaging and social media apps as tools for qualitative research. Building upon our research on Italian migration to Shanghai, we discuss in detail the methodological choice of using WeChat for teamwork, remote sampling strategies, and conducting interviews. The paper highlights the benefits that researchers may have from employing the same technology that the studied community uses in their daily life as a research tool, and advocates for a flexible approach to research that adapts its tools and methods to the specific requirements and characteristics of the fieldwork. In our case, this strategy allowed us to emphasize that WeChat represents a digital migratory space which played a crucial role in understanding and making of the Italian *digital diaspora* in China.

## Introduction

This paper presents a methodological reflection based on our studies on the Italian community in Shanghai and focuses on the potential and limitations of using WeChat as an investigation tool for qualitative research. Our studies on the Italian immigrants in Shanghai began in 2018 as a qualitative investigation in the overall research project of the Documentation Center on New Migrations of the University of Salerno (Ce.Do.M.-UNISA). We used WeChat as the main tool for conducting our research and carry out interviews. WeChat is the leading internet communication system in China, offering traditional messaging, multimedia sharing, and represents a key element in the everyday life both for Chinese people and for the immigrant population in China. This tool was key in recruiting interviewees and creating relationship with local key informants, who were representatives of associations of Italians abroad or spokespersons for Italian cultural centers in Shanghai. On the basis of these studies, we analyze the benefits of using WeChat as a research tool. First, it helps researchers to create an informal environment where they can have frequent and real-time feedback that facilitates communication. Second, as we obtained the personal phone number or WeChat ID of key informants in Shanghai, almost all other communications were held online. This enabled us to implement a strategy of snowball sampling that allowed us to effectively reach a great number of people remotely and at no cost. Moreover, instant text and audio messages make easy to have asynchronous communications, that is key when working in different time zones. Third, as for other web-based tools, audio and video interviews are free, easy to use and familiar to interviewees who only need their mobile phone. Fourth, since WeChat is a ubiquitous tool in the interlocutors' daily life, used for both professional and social purposes, this tool allowed us to gain deeper insight into the *digital lifeworld* of the community studied. In fact, Italian immigrants in China regularly update their WeChat profiles and share information, feelings, and ideas with friends and public. Having access to their digital space, we gained valuable insights into their daily habits, social activities, emerging problems, and work conditions. This provided us with access to a wide variety of supplementary material, which helped us analyze the interviews upon which the study was based. However, the large amount and the variety of this material prompted us to consider how best to utilize it for our research purposes. This poses a stimulating methodological challenge, which drives us to go beyond using WeChat as a data collection tool and reconceptualize it as a social object of enquiry to be explored using the netnographic approach. This shift in research method, which does not rely merely on the planned interviews, also poses practical and ethical challenges in terms of informed consent, data collection and analysis. These opportunities and limitations are discussed in the section “Challenges and lessons learned”.

In the conclusions, the paper highlights the benefits that researchers may have in using the same communication tools that the examined community itself uses to build and maintain social relationships in their everyday life. It advocates for a methodology that is responsive to the unique needs and circumstances of each specific case study. This approach prioritizes the subjects' involvement in the research by avoiding the imposition of tools that only suit to the researcher's habits and needs.

This encourages us to make sense of the specific use that Italian community in Shanghai make of WeChat as a digital migratory space, thereby contributing to the broader debate on the digital diaspora.

## WeChat in the context of the Italian migration to Shanghai

The most popular Internet-based communication system in China is Wēixìn (微信), launched in 2011 by the Chinese technology company Tencent. Since 2012, WeChat has been available globally as an international version of the original Wēixìn; the two are interoperable. For simplicity, in this paper we refer to both as “WeChat”. Like WhatsApp, WeChat integrates the most classic messaging and multimedia sharing services with features that allow users to create and manage multiple groups, laying the foundations for the establishment of online communities. In addition, WeChat has a public message board for sharing images and short videos (Channel) and social networking features (Moments), which replace social media such as Instagram or Facebook. It offers mobile payment services (paying fines, booking taxis, food delivery, bicycle rental, etc.) with the possibility of starting and managing a business. With WeChat “City Services” you can also book doctor appointments, report incidents to the police, control traffic, and much more. All these features encourage users in China to use WeChat on a regular basis, whether for business, entertainment, or everyday social interactions with family and friends. The attachment of Chinese users to WeChat is such that Chen et al. (2018) define the app as “Super-Sticky”.

WeChat has been properly described as a ≪digital Swiss Army knife for modern life≫ for Chinese users (Lee, 2018, p. 17; Miller et al., 2021, p. 84). Recent literature has also highlighted that ≪WeChat is an essential part of the migration infrastructure≫ for both Chinese migrants throughout the world and foreign immigrant populations in China, since it compels them to live in the Chinese digital world (Ryzhova and Koreshkova, 2022).

Because the Chinese government restricts many Western social media, without WeChat immigrants would face not only marginalization in China, but also increased difficulties in maintaining relations with relatives and affections in homeland and around the world. On the one hand, using WeChat for immigrants is the most comfortable alternative if not a forced choice. On the other hand, migration to the Chinese digital world is not a neutral process. For Western people it contemplates a radical change in the cognitive landscape, which is affected by the ideological and value conflict between the two societies.^1^

Our studies on the Italian immigrants in Shanghai began in 2018 as a qualitative investigation in the overall research project of the Documentation Center on New Migrations of the University of Salerno (Ce.Do.M.-UNISA). The main aims were: (i) to outline the profiles of Italians who decide to migrate to the Chinese metropolis of Shanghai; (ii) to understand the factors that influenced this decision; (iii) to collect stories and experiences of Italians in China to identify the opportunities and constraints they are experiencing in the new environment.

A significant part of the research was planned and completed before the outbreak of the COVID-19 pandemic. The empirical basis was obtained through individual semi-structured qualitative interviews *via* videoconference or audio-only call. We recruited 53 interviewees (28 men and 25 women) through a snowball sampling procedure from a core of local key informants, who were representatives of associations of Italians abroad or spokespersons for Italian cultural centers in Shanghai. Over time, we have maintained continuous contact with respondents and reached new ones.

After a few years, in spring 2021 we launched a new study on the impact of the COVID-19 pandemic on the migration of Italians to Shanghai. This focus involved 20 key informants, who responded to open-ended questions in writing. The network of relationships we have built with individuals and associations of Italians in Shanghai allows us to imagine the continuation of the research with longitudinal studies, enrolling new participants.

The research team included three researchers based in Italy and one research assistant in Shanghai. The scientific director was in Italy as well. Structuring fieldwork with the relocation of a portion of the team to Shanghai for a long period of time would not have been viable, both economically and because of career and personal responsibilities. There was the option of conducting face-to-face interviews entrusting the entire task to the Shanghai research assistant. However, such a strategy would hardly have laid the foundation for an ongoing relationship with respondents, which was necessary to observe changes over time. For this reason, we had assumed to carry out our study with interviews remotely by employing five more interviewers recruited and trained in Italy. In addition, because to the unexpected pandemic situation, face-to-face interviews would have been problematic.

Due to the peculiar characteristics of the population, the choice of a smartphone app to interact with them would have guaranteed more advantages than disadvantages. The research takes place in Shanghai, a metropolis with high levels of mobile penetration, and focuses on a young and cosmopolitan community of people who need to maintain their network of relationships in Italy and the world. Because government in China is interested in attracting skilled talent to support social and economic development in the country,^2^ immigrants from Italy are generally highly educated professionals.

We needed a communication system that would allow us to make low-cost international calls and send messages, both synchronously and asynchronously, to easily communicate with people located in different time zones. It was crucial to allow them to participate in comfort, with a familiar and flexible tool, respecting their life and work times. We also considered that using an instant messaging app could make communication faster and more conversational than email (see Hinchcliffe and Gavin, 2009), strengthening bonds with respondents and thus reducing the risk of dropout.

Although in 2018 the literature on the use of WeChat in social research was scarce, we were aware of the increasing use of it to recruit participants for questionnaires or in-depth interviews in China (Montag et al., 2018). In just a few years, WeChat has become one of the most popular smartphone apps in China, not only for the size of the community but also for the increase in function with “mini-programs”.

“Here we only use WeChat and Alipay to pay. With WeChat you can pay for the taxi, too.” (I01).^3^

Since the outbreak of the coronavirus pandemic, WeChat has also been used to obtain and carry the “Health Code” (the Chinese digital pass). COVID-19 vaccination and test records can also be checked on WeChat (Liang, 2020).

“On WeChat you have your own health code, which is a sort of traffic light. You must hope every day that it is green! Green means you are free; otherwise… well, better stay at home.” (I02).

From the information gathered in the preliminary phase of the research and the personal experience of the research assistant in Shanghai, WeChat seemed to be the best choice. Subsequently, all the respondents confirmed that they prefer WeChat for their inside and outside China communications. We observed that Italian immigrants use WeChat to actively engage in Shanghai's Italian community and maintain relationships with family in Italy and Italians around the world (Moffa and Chirivì, 2021; Moffa, 2022).

“To communicate with friends and family who are not here in Shanghai, some still use Skype. But it is very rare: WeChat is more comfortable. I only use Skype with my husband's older parents… but as soon as we go to Italy, I will sign them up on WeChat.” (I03).

“I miss Italy. I have a fridge full of San Daniele ham! Luckily, with WeChat I am in touch with my world every day. I have many WeChat groups with family and friends. I have the group for the favorite team, the one for travel, the one for recipes and traditional dishes, or whatever. You can share photos and videos, you can joke … You are together! Here all this would not be possible without WeChat. When I am in Italy, I also use WhatsApp, but it does not allow me to stay in touch with friends in China. WeChat connected us.” (I04).

Moreover, it seems that WeChat is also favored for institutional communications of Italian associations in Shanghai.

“We have a Facebook page, but we have not updated it for a long time. We use WeChat.” (I05).

“Now we only use WeChat… for everything. There is also a WeChat group for [the Italian association of migrants in Shanghai]. We are 150 members.” (I06).

## WeChat as a tool for online interviews: Methodological considerations

Using WeChat with people living in China for social research offers many advantages and opportunities, but it also has constraints and disadvantages. Some of these pros and cons are presented in the following paragraphs. We will also distinguish between what we had planned and what emerged from the field work. In fact, our evolving experience with WeChat and its users has pushed us to move the research in unexpected directions.

A private group has been created on WeChat to have frequent, real-time feedback among team members in an atmosphere of informality that facilitates communication (see Jailobaev et al., 2021). The private group was also a “protected space” to acquire familiarity with WeChat and experiment with its various tools. Almost all the members of the research team have used the app for the first time and the research assistant has guided us toward a conscious and efficient use.

We preferred to avoid using WeChat to share sensitive documents, such as the outline of semi-structured interviews and the transcripts, to avoid the risk of banning. It is well known that the Chinese government controls and censors the material that circulates on WeChat (see Sun and Yu, 2022, p. 260). There is proof that they run an algorithm to track and ban posts and accounts that use sensitive keywords (Ruan et al., 2016). In fact, a team member was banned from WeChat after submitting a draft question on the impact of COVID-19 and China's lockdown strategy on Italian migration.

The initial contact with key informants was made by our research assistant in Shanghai. This interaction usually took place face-to-face; subsequently, almost all other communications were held online.^4^ Once their personal or institutional phone number or WeChat id was obtained, the scientific director contacted them on WeChat. Through text messages, the scientific director explained the purposes of the research, and collected suggestions about the topics to explore and the people to contact. In this way, the key informants were actively involved in research, entrusting them with a privileged role in a sort of “collaboration agreement”. Most of the key informants gladly accepted this agreement and are still collaborating in the development of the research.

Key informants played a leading role at the beginning of the study. After analyzing the relevant scientific literature on the topic, we needed to learn more about the emerging issues and the current living conditions of the Italian people in Shanghai. The specific objective was to clarify the analytical parameters on which to build the outline for subsequent semi-structured interviews. From their privileged point of view, local key informants suggested new themes and little-explored aspects of the Italian migration phenomenon. In addition, key informants were the starting point for the sampling plan according to a snowball strategy.

WeChat has been particularly useful in recruiting potential respondents thanks to the digital “name card” feature for sharing contacts. By submitting the name card, a person can share his or her contacts with another WeChat user if both are on his or her WeChat contact list. The name card includes public profile information, such as username, profile picture, possibly real name, gender, telephone number or email address. Furthermore, the sender can add a few lines of presentation. Only fifty characters are allowed, including spaces.

Those who receive the name card can send a “friend request”; by approving the request, the person contacted opens a new communication channel on WeChat. Similarly, knowing the username or mobile number, you can send any user a friend request attaching a brief introduction to your contact details. We have always attached the official link to provide all the information about the context and purpose of the study. In this regard, we found the importance of building a solid digital identity for the team and individual researchers both in WeChat and on websites that people can reach independently and at any time.

We were surprised to see that some of our primary informants have set up group chats to introduce us to their contacts. For instance, the presidents of cultural circles frequently introduced us to their colleagues in the association's main group or by opening a new one for that purpose. The desire to share experiences and be heard has made these chat groups similar to “spontaneous” online focus groups. This provides good opportunities to collect textual and relational data for further analysis.

Thanks to these online interactions, we were able to reach many Italian immigrants in Shanghai, improving our sampling. The disadvantage of relying excessively on a WeChat-based sampling strategy is that owning an account and knowing how to use it is a barrier that prevents us from reaching people who are less technologically informed or who do not use WeChat. But this is, likely, an unusual condition among the subjects of our interest. It is worth saying that they were free to communicate with us in other ways, but this never happened.

Online text messages are used for both the initial contact and for scheduling the interview. A significant advantage of asynchronous communication is that ≪the availability of a persistent textual record of the conversation renders the interaction cognitively manageable, hence offsetting the major “negative” effect of incoherence in spoken interaction≫ (Herring, 1999). It also helps to overcome the barrier due to the different time zone in the delicate recruitment phase (O'Connor and Madge, 2016, p. 417).

Once availability is achieved, the scientific director introduces the interviewer and respondent to each other *via* the “name card” tool. At this stage, it is made clear that the interview requires the right attention, with a specially dedicated time and space, so it is necessary to make an appointment. Then, interviewer and interviewee reach an agreement *via* WeChat.

We preferred to use email rather than WeChat to confirm the date and obtain informed consent. The email communication sent from the institutional account guarantees greater formality to the interview request. The email also allows us to attach important documents without triggering WeChat's censorship algorithm. Together with the leaflet that reminds them, once again, of the context and purposes of the study, we deemed it appropriate to anticipate in writing the questions we would ask. In accordance with agreements, the interview took place with an audio or video call on WeChat.

Right after the interview, the scientific director contacted the interviewee *via* WeChat to thank him. This was a key step because it allowed us to take care of the relationship with the interviewee and keep open a communication channel for further reflections and future insights. Later, the transcript was emailed to each interviewee to obtain confirmation about the fidelity of the data and the release for use. This method gives respondents the opportunity to better clarify their point of view, correct any mistakes, or add additional considerations that they feel are worthy of attention. In this way we ensure that the text analyzed and presented in the research results has been approved by the actors involved.

The main advantage of phone interviewing through WeChat is that it is free, easy to use, and familiar to respondents. The call works over the internet and can be taken from both a smartphone and a computer. Using WeChat, we avoid asking them to download and install additional software, sign up for an account, and learn how to use a new tool. It should also be considered that using non-Chinese software in China, such as Microsoft Teams, can be complicated due to government restrictions. Getting around these blocks requires technical skills that we could not take for granted.^5^

One downside is that WeChat, unlike Microsoft Teams or Google Meet, does not have a built-in feature to record and transcribe the call. After obtaining explicit consent to registration, we had to proceed with an external device. Since respondents mainly used WeChat on their smartphones, the second downside concerns the multiple distracting factors for notifications or phone calls that came in during the interview. Based on our experience in the field, we assume synchronous interviews *via* WeChat (or other smartphone apps) are more prone to interruptions or postponements due to interference than face-to-face interviews. The third downside is that most of the time the video call turned into an audio-only call, excluding the possibility of observing body language and the environment.

The second phase of the research followed a different method. The focus on the pandemic was possible precisely because, about a year after the end of the previous study, a digital community of Italians residing in China had already been formed around the research team. From this community, through one-to-one messages or group chats on WeChat, the need emerged to investigate the impact of the pandemic crisis on Italian immigrants in China.

For exploratory purposes, emerging issues were first covered in short individual interviews on WeChat *via* instant messages or short audio recordings. Then, after establishing the research questions and the key concepts, we recruited a panel of informants and sent them a semi-structured interview with written and open answers. To the spontaneity of interactions on WeChat, we have associated a type of interview that produces authentic texts with greater reflexivity. In both cases, the advantage is to collect the written text directly without the need for transcription.

## Beyond the interviews: Insights and supplementary material from WeChat

As anticipated, throughout the long duration of the research we managed interactions with our respondents and key informants through WeChat. In this way, we have been able to get closer to the *digital lifeworld* of our interlocutors, who use this tool daily and pervasively both for work and social life.

Although the specific object of our studies was not the use that Italian migrants make of WeChat, respondents often brought this theme to our attention. They told us how it is helpful for shopping, organizing trips, promoting cultural or leisure activities, or simply exchanging information, photographs, videos, and documents: “I don't really know how to do without it.” (I07).

Using an instant messaging tool facilitated the construction of a mutual and fruitful relationship between the researcher and the subject of the investigation. Over time, this digital relationship has produced much more material than we requested and expected. By “expected material”, we mean what we collected according to the research project. It is basically about records and texts we obtain from online interviews and notes from the interviewers.

As mentioned, the interviews were conducted by trained interviewers; the other contacts, before and after the interview, were maintained by the scientific director. During these interactions on WeChat, respondents and key informants spontaneously sent us a lot of material. These are text messages, web links, photos, videos, and digital documents that deepen their opinions on the topics of the interview but also GIFs, emojis, and memes. In this mass of this “supplementary material”, we distinguish between what respondents themselves have produced and the material taken from the web or from other WeChat users.

The first set of materials includes photographs they took “in real time” during a conversation to share with us their life in China. This often happened as an answer to a question from the researcher, not only during the actual interview but also in the interactions before and after the interview. Taken as a whole, these photographs constitute a valuable archive that can be explored through the lens of visual sociology.

We interpret these events as a sign of respondents' involvement in the research. It is also a desirable byproduct of employing a versatile tool that offers the interviewee several opportunities to respond to a stimulus beyond the simple text or audio message. In addition, because chat messages are recorded and communication proceeds asynchronously, the door for new interactions is always open.

In particular, those who are part of associations or clubs continue to share with us the appointments, initiatives, and publications of their community. At their invitation, we are still subscribed to the WeChat channels of these people and organizations.

The possibility of exploring a large amount of material published on personal and institutional WeChat channels of Italians in China suggests a further distinction between “direct” and “indirect” material. By “direct” we mean what the subject has sent us firsthand and voluntarily. By “indirect material” we refer to everything that constitutes the digital trace of the subject and is independent of our intervention.

WeChat users, including our interlocutors, curate their profiles by updating their photographs and “status” messages; through Moments, they share emotions and information with their existing friends, who respond with *liking expression* (Chen et al., 2019; Li and Wang, 2021); they interact with small or large groups or share their ideas with the public in Channels. By exploring WeChat, we learn about their daily habits, worries, joys, and emerging social problems. Following the personal and club channels, we find posters, photos, videos, and other material that tell us a lot about what social and cultural activities are organized by Italians in China, what is the level of involvement, and what is the mood of the participants. We also find out about social problems, working conditions, and career opportunities. Precisely the wealth of “supplemental” and “indirect” material that is available in the digital world that Italian migrants in China build on WeChat allows us to understand more deeply the reality of their daily lives. In short, thanks to WeChat, we can participate in the same Chinese (digital) world in which our interlocutors live, regardless of where we are physically.

Especially in reference to the pandemic, this method has allowed us to access a distant and otherwise impenetrable world. A world that seems alien to us but for the subjects of our research is everyday life.

During the early stages of the pandemic, key informants kept us updated through WeChat messages about the situation in Shanghai. In mid-January 2020, along with sending us GIFs of greetings, several messages expressed worries about the potential spread of the new virus during the Chinese New Year celebrations. On January 24th, some sources shared their concerns about COVID-19 and lockdown.

In February, through WeChat, we were engaged in the initiatives to send our compatriots in Shanghai masks for personal protection from contagion. From key informants, we receive reassuring news about the Chinese government's ability to contain the spread of the infection and its economic impact. The stories about the new difficulties faced by foreigners in Shanghai were more worrying, especially for the “broken families” between China and the homeland.^6^ In the following year, some messages underlined the progressive decline in the living conditions of Italian migrants in Shanghai.

*The situation for the Italians who are still here has totally changed. “Bad winds are blowing”. Many are leaving. It is not just about lockdown, quarantine, and prevention measures. The price of airline tickets goes up… Furthermore, there is the problem of vaccination…*

On the one hand, supplementary material lies outside the original research design; on the other hand, it provides us with a deeper understanding of our research object, allowing us to formulate new challenging questions. To enhance this material and experiences on the (digital) ground, we are called to modify the research design by assuming a *netnographic* position (Kozinets, 2015).

## Challenges and lessons learned

Because it was simple, multimedia, and conversational, using WeChat pushed us beyond what we had planned. One of the strengths of using WeChat for our qualitative social research—even just concerning solely interview-based study—is that our ability to analyze respondents' stories improved in empathic understanding through a process of *perduction* (Piasere, 2002). The research team had the opportunity to attend continuously and for a long time a part of the digital world of which the subjects of the study are inhabitants and creators. Of course, as vast as this digital world is, it is only a little window into the world of Italian migrants in Shanghai. However, inhabiting a common space on WeChat has allowed us to internalize—more or less consciously—some cognitive-experiential patterns of our interlocutors and, therefore, to better understand their narratives, problems, expectations, and hopes. In this sense, we were able to deepen and integrate the online interviews (audio, video, and text-based) with a form of online participant-observation.

The second strong point is that the attendance of our informants on WeChat has allowed the building of a sort of panel that we can easily engage for further studies in a longitudinal approach. The availability of a large group of informants, easily reachable *via* smartphone, allows us to activate new interview-based studies or to draw information from their digital track on WeChat. The key informants themselves, who are leading figures of the Italian community in Shanghai, urge us to continue the research and expand it to new themes. Already in 2021, after sharing the reports of the first phase of the research, the common need to continue research with a focus on the pandemic emerged. Today the informants themselves insist on the need for in-depth analysis, since the feeling is that the recent change in the working and living conditions of Italian migrants in China is not only linked to the pandemic crisis. Rather, the pandemic may have been a catalyst for transformations already underway in the Chinese government's migration policies (Moffa, 2022).

“The living conditions of the Italians here are changing, and it is not just due to COVID. You should conduct an in-depth study.” (I08).

“We need a new study; things are changing fast.” (I09).

“An interesting topic to explore would be the choice of many Italians to move to inland areas of China.” (I10).

The main weakness is that we have not used the full potential of WeChat as a tool for collecting data and as an online environment to be studied with a well-structured netnographic approach.

The classification and analysis of the large amount of supplementary material we collected requires a specific methodology. There are several paths we could take for the continuation of the research. Below we hypothesize some of them, drawing inspiration from the relevant literature on the topic.

As anticipated, we participated in several group chats frequented by Italians living in Shanghai. In some cases, these were already existing groups, for example, those of Italian migrant associations or circles active in Shanghai; in other cases, they were groups created by key informants specifically to introduce us to other people to interview. Among other themes, some topics relevant to our research are spontaneously discussed in these groups. Interesting discussions on WeChat also develop in the comments to the Moments and posts in the Channels. Submitting all these contents for analysis and disseminating the results would be stimulating. Nonetheless, questions of a pragmatic and ethical nature arise.

To constitute a relevant corpus for analysis, the whole text must first be subjected to a long and complex cleaning operation to select only the contents congruent with our research interests, eliminating what does not concern us. Then, there is the delicate problem of privacy and the collection of consent from many interlocutors regarding texts and other contents they produced for purposes other than our research (see Varnhagen et al., 2005).

For the online interviews, consent was explicit, and agreement to use the information they produced for research purposes was confirmed at multiple stages, before, during, and after the interview. In spontaneous interactions, both group and one-to-one, explicit consent may be missing.

In the case of direct interactions based on text or other authentic content, one might assume that consent is implied since that person has been adequately informed from the outset that we are conducting social research. This already questionable assumption becomes untenable in the case of interactions on WeChat groups and Channels.

Whenever possible, we preferred to always ask for explicit consent from the interested party; otherwise, we have refrained from publishing those materials. The same applies to excerpts included in this paper and in previous publications from the studies (Moffa, 2022). However, systematically gathering user consent for comments and other interactions on WeChat would be far-fetched.

Accepting the limitations of doing social research in a less “natural” and “spontaneous” setting, a possible alternative is to conduct asynchronously an online focus group. The method is to invite a select group of informants to join a WeChat group we created to discuss a research topic.

The advantages and disadvantages of the online focus group method with instant messaging apps are recently discussed in Jailobaev et al. (2021) and Neo et al. (2022). For further information on online focus group, we refer to Murray (1997), Mann and Stewart (2000), Abrams and Gaiser (2016), and Barbour and Morgan (2017). Here we limit ourselves to pointing out that online focus groups on WeChat are low cost, relatively easy to moderate and manage, mainly produces text-based interactions that we can analyze without having to transcribe. The asynchronous mode would allow everyone to participate from different time zones and take the time to read and reflect (as long as the chat is well coordinated). Participants, possibly encouraged by the moderator, can also share multimedia content like videos or photographs. The release for the use of anything shared on the group is collected from the beginning explicitly; however, respecting privacy remains a sensitive issue as anonymity on WeChat cannot be guaranteed by the research team. The entire discussion is recorded on the participants' devices: the research team cannot control the dissemination of all or part of it to third parties. Another delicate issue is the risk of incurring a ban if topics or keywords unwelcome to WeChat's censorship algorithms emerge in the discussion.

The second direction of research requires a deeper immersion in the digital environment inhabited by Italians in Shanghai. In studies based on interviews or focus groups, the researchers are clearly defined in the role of external subjects interested in listening to learn more about their research subject. They ask questions or introduce discussion topics. In our case, the research team, with a particular focus on the scientific director, was called to participate more and more actively in the social dynamics of the Italian community in Shanghai. Almost all the interactions we participated in, took place on and through WeChat in an informal environment that favored a more conversational and informal approach, even in relationships between strangers.

A clear sign of the transformation of the relationship in the direction of greater closeness between the respondents and the scientific director, in particular, is that the formal linguistic register soon gave way to more direct communication. Here is an example of this process. When speaking Italian with someone you do not know well, it is common practice to address the other in the third person, using the pronoun “lei” instead of “tu”. It was common for our respondents on WeChat to prefer informal language and to switch to second person during conversations, particularly for those cosmopolitan subjects who are more comfortable speaking English in everyday situations and less prone to use formal Italian writing.^7^

Another sign is that voice messages, emoticons, and other visual content have increasingly accompanied text messages as the relationship progressed. In this way, the story that Italian migrants tell us about their life in Shanghai is enriched with content and depth, in new directions compared to what the interviews alone tell. Often, they send us photographs taken “in real time” or videos or other documents that testify to situations and events that are significant to them. Their intent was to involve us intensely in the narration of the daily reality of Italian immigrants in Shanghai, suggesting the keys to reading a complicated world that they perceived as immanent and distant at the same time.

Italian migrants shared with us photos of their travels, along the Shanghai Metro and beyond. We saw where they work, the magnificence of the skyscrapers, and the charm of the old part of the city through the cameras of their smartphones. Even if at a distance, we experienced with them the dramatic beginning of the pandemic ahead of the rest of the world. On WeChat, we follow the same people watching their Moments, participate in the same Channels and group chats, and keep up with the news that affects them but were unfamiliar to the majority of Italians.

Through the lens of our Italian migrants in Shanghai, we have been able to observe some phenomena as they have occurred, even earlier than the rest of the world in the case of the spread of the COVID-19. It would have been difficult if we had limited ourselves to interviews. In short, because the daily lives of Italians in Shanghai include online exchanges on WeChat, we were a part of their lives for nearly 4 years (see Hallett and Barber, 2014).

The interaction has gone beyond the conventional roles of observer and observed on several occasions. Some of the people observed, who are in charge of organizations, academics, or Ph.D. candidates, approached us for advice and support in their social research, recognizing our role as experts. They have made us participate in their research objectives, sharing projects and study materials on various topics concerning the life of Italians in China, from everyday life to industrial issues. Other times, they have turned to us to seek solutions to the practical problems of the Italian community in Shanghai. In this type of relationship, the observer turns into a resource and a point of reference outside the community. This happens precisely because of the distance and the academic role covered by the researcher, who is entrusted with the task of interceding for them from Italy.

This has happened to a greater extent with the advent of the pandemic when new problems and needs emerged among Italians in Shanghai. An example is the difficulty in supplying mask to the community during the initial phase of the pandemic; another is the need for support to address limitations in international travel, which have divided families and put professionals and businesses in serious difficulty. Due to the complicated setting in which the interaction took place, which is typical of participant observation, we interpreted the findings reflexively, by taking into account our dual placement inside and outside the group studied.

This fully immersive experience helps us to shift our mental models toward a more global and less Eurocentric outlook. From all these considerations emerges the opportunity to pursue research in the direction of a well-structured netnographic approach (Kozinets, 2015; Kozinets and Gambetti, 2021) which complements our broader methodological framework. By shifting the level of analysis from the discursive productions of individuals to the digital social relations within the social aggregation ≪that emerge from the net≫ (Rheingold, 1993, p. 5), the aim is to focus on the implications of using WeChat for the digital diaspora of Italians in Shanghai in terms of identity building and social inclusion (digital and not) of the migrants in the host country.

In recent literature, we find some studies on the use of WeChat in the Chinese diaspora (Sun and Yu, 2022); rare are those on the use of WeChat by immigrants in China (Ryzhova and Koreshkova, 2022). By continuing our investigation, we intend also to contribute to the effort to ≪de-Westernize platform studies≫ (Davis and Xiao, 2021).

## Conclusion

A rich literature on migratory phenomena has long underlined how the geography of migration has drastically changed in the international and global context, and how nowadays references to national borders are deceptive and outdated. In the Age of Migration (Castles and Miller, 2009), the routes of migration intersect, and the variegated socioeconomic compositions of individuals participating move within a setting where outflows, inflows, and transit flows coexist (Calvanese, 1992; Ambrosini, 2019). Within the broad debate on the topic, several studies now focus on *transnationalization* going beyond the conventional country of arrival/country of departure distinction and widening our perspectives on the topic (Glick Schiller et al., 1992; Vertovec, 1999; Faist, 2000; Ambrosini, 2008). Furthermore, scholars focusing on *digital migration* (Komito, 2011; Dekker and Engbersen, 2014; Kok and Rogers, 2017) and *digital diasporic groups* (Hiller and Franz, 2004; Alonso and Oiarzabal, 2010; Laguerre, 2010) are increasingly finding a voice in migration studies.

It is within this framework that this paper contributes to the debate on the new Italian emigration in Shanghai from a methodological perspective that takes into account the interactions produced on WeChat. With reference to our fieldwork, Shanghai is perceived by the migrants interviewed as a Megalopolis connoted by a liquid modernity (Bauman, 2000), in which they struggle to find a social and collective dimension typical of public places. According to what emerged from the analysis of the interviews, Shanghai is perceived as a ≪non-place≫ (Augé, 1992): ≪defined by stereotyped spaces, lacking relationships and frequented by groups of people in transit≫ (Moffa and Chirivì, 2021), who consequently live in a socially floating setting.

With the words of Ambrosini (2008), we have encountered *transmigrants* who conceive migration as a fluid, non-territorialized process. They nevertheless maintain ≪across borders a wide arc of social relations≫ (p. 45) thanks to the technological mediation of WeChat. As previously mentioned, this platform constitutes a powerful means to networking personal and professional relationship, experienced in a social space that is not conditioned by national borders. We argue that the contacts created in these particular digital settings lead to the creation of new types of virtual and international communities.

This further consolidates the dimension of transnationalism in which ≪the here and the there are thus perceived as complementary dimensions of a single space of experience≫ (Ambrosini, 2008, p. 48). WeChat is a tool that makes communication deep, dense, instantaneous, continuous, and replicable and most importantly, helps to the breaking down of geographical borders by fostering the multidimensionality of the cosmopolitan migrant's experience. Through the analysis of the interviews collected, we realized how much this platform supports and encourages an ≪imagined community≫ (Georgiou, 2006) that, starting from the members of the physical diaspora and their families, spreads beyond.

From this perspective, it can be argued that the new Italian emigration to Shanghai encountered in our studies is comparable to what some experts on migration call the ≪digital diaspora≫ (Laguerre, 2010; Andersson, 2019). Indeed, it presents its three ≪building blocks≫, namely: (i) it is a migratory flow, (ii) it makes extensive use of information technology, and (iii) it shares information and weaves networks (Laguerre, 2010).

According to this line of studies, the strength of the digital diaspora lies in the possibility of recreating shared spaces in which ≪receive support in understanding and expressing the homeland culture and negotiate hybrid identities≫ (Brinkerhoff, 2009, p. 82). This is exactly what we found during our investigation, to the point that we ourselves, as researchers, have become—at least in part—members of that community, observing from the inside how Italian migrants in Shanghai deal with emergencies but also with ordinary problems due to the differences in culture and customs between the country of origin and the country of arrival.

Therefore, we argued that the interviewees inhabit a *digital migratory space*^8^ as a counterbalance to the *non-place* dimension they experience in Shanghai. In other words, the digital dimension allows them to free themselves from physical places and connect with other realities. It is in this space that the multidimensionality of the migration experience is realized. In this sense, the digital reality represents a *place-localized*, albeit diverse and fragmented. Through the WeChat tool, the interviewees find themselves inhabiting a space nurtured by connection practices, by specific proximity relations, in response to their need to hold together different universes (Italy, Shanghai and the relationships between them).

Tying up the knots of the proposed reasoning, we observe that on WeChat ≪multiple belongings≫ (Rocha-Trindade, 1990; Calvanese, 2000) take shape and consolidate as well as the different integration paths of our interviewees. Moreover, as Sun and Yu (2022) point out, for migrants and diasporic communities, digital communication technologies have become an indispensable dimension. For this reason, it is believed that research on the *digital migratory space* offers an opportunity to take a closer look at migratory practices. From this perspective, the study of interactions developed through digital media, which themselves become “places”, appears essential for understanding the dynamics of modern diasporas and, more generally, the processes of social inclusion or, on the contrary, exclusion of migration.

## Data availability statement

The raw data supporting the conclusions of this article will be made available by the authors, without undue reservation.

## Ethics statement

Ethical review and approval was not required for the study on human participants in accordance with the local legislation and institutional requirements. The patients/participants provided their written informed consent to participate in this study.

## Author contributions

GM and MD contributed to conception and design of the study. All authors contributed to manuscript revision, read, and approved the submitted version.

## Footnotes

^1^In this regard, Miller et al. (2021) describes the “red envelope” and “kinship card” functions as examples of incorporating Chinese culture and traditions into WeChat. Using these app features, foreign users are socialized to Chinese culture.

^2^Local authorities in Shanghai, especially, have undertaken special initiatives for skilled immigration (Centre for China Globalization, 2017; Tao et al., 2022).

^3^The excerpts from conversations and interviews with Italian immigrants in Shanghai are translated into English from Italian. In this paper, we have marked them with the letter I followed by a number in order of appearance.

^4^Before COVID, more than one interlocutor invited us to be their guests in Shanghai or to meet them in Italy. When the meeting took place, it was an opportunity to strengthen the bond and further expand the network of contacts.

^5^You can access social media and government-censored apps from China using virtual private networks (VPNs).

^6^For an overview on the study of *transnational family* see Madianou and Miller (2011), Madianou and Miller (2013), and Kilkey and Palenga-Möllenbeck (2016).

^7^We also see a general tendency to use a formal linguistic register in (rare) email communications, even when communication on WeChat was informal.

^8^The reference is to the concept of ≪migratory space≫ introduced by Simon (1979). For *migratory space* we mean the spatial dimension defined by the set of bonds that the protagonists of the migratory process constitute in their social relations in and across countries.
